# Supplementary material for: Explicit and exact travelling wave solutions for Hirota equation and computerized mechanization
Source: PLoS One. 2024 May 21;19(5):e0303982. doi: 10.1371/journal.pone.0303982 (PMC11108183; doi:10.1371/journal.pone.0303982)
Supplement: Attachment — Submitted filename: Comments on PONE-D-24-02488.pdf [file pone.0303982.s001.pdf]

Review for PONE-D-24-02488

The research work focuses on finding explicit and exact travelling wave solutions for the Hirota equation through various mathematical methods. The paper presents a significant contribution by introducing new solutions for the Hirota equation, enhancing the understanding of nonlinear physical phenomena. Here are some suggestions for this article:

1. How do the chosen methods (power-exponential function method and the extended hyperbolic auxiliary equation method) compare in efficiency and accuracy with other contemporary approaches to solving the Hirota equation?

2. Can the authors elaborate on the unique aspects of the solutions found for the Hirota equation and how these contribute to advancing the field?

3. What are the theoretical implications of the new solutions for understanding complex systems modeled by the Hirota equation?

4. Are there specific practical applications in physics or engineering where the new solutions could be particularly beneficial? How do these solutions improve upon existing models or methodologies in practical scenarios?

5. What are the limitations of the current study, and what assumptions have been made in the application of the methods? How do these limitations affect the interpretation of the results?

6. Based on the findings, what future research directions do the authors

suggest? Are there any particular areas within the field where these solutions could open up new lines of inquiry?

7. Could the authors provide more detail on the data or simulations used to verify the solutions, ensuring reproducibility for other researchers?

8. How do the solutions derived in this study perform in comparison to existing solutions to the Hirota equation in terms of stability, solvability, and physical relevance?

Overall, the paper makes contributions to the field of nonlinear partial differential equations, particularly in the study of the Hirota equation. Expanding on certain aspects as suggested could make the paper even more robust and impactful.
